# Nutritionally Improved Gluten-Free Breads Fortified with Soluble Fiber and Bioactive Compounds from Artichoke and Broccoli By-Products

**DOI:** 10.3390/molecules31010152

**Published:** 2026-01-01

**Authors:** Jhazmin Quizhpe, Rocío Peñalver, Pablo Ayuso, Gema Nieto

**Affiliations:** Department of Food Technology, Food Science and Nutrition, Faculty of Veterinary Sciences, Regional Campus of International Excellence “Campus Mare Nostrum”, University of Murcia, Espinardo, 30100 Murcia, Spain; jhazminedith.quizhper@um.es (J.Q.); rocio.penalver@um.es (R.P.); pablo.ayuson@um.es (P.A.)

**Keywords:** functional food, gluten-free bread, food by-products, enzymatic treatment, dietary fiber, digestible starch

## Abstract

Background: Commercial gluten-free (GF) breads often exhibit low nutritional quality due to limited fiber and bioactive compounds. The enzymatic treatment of vegetable by-products, such as broccoli and artichoke, represents a sustainable strategy to release soluble dietary fiber and phenolic compounds, enhancing the functional value of GF products. Five GF bread formulations were developed: a control bread, breads containing broccoli or artichoke extracts (BB and BA), and breads with enzymatically treated extracts using Viscozyme^®^ L and Celluclast^®^ 1.5 L (BBE and BAE). A commercial GF bread (BC) served as a reference. Nutritional composition, dietary fiber fractions, phenolic content, antioxidant capacity, starch digestibility, physicochemical parameters, and sensory properties were evaluated. Results: Enzymatic treatments significantly improved the nutritional and functional properties of GF breads. Viscozyme L^®^ produced the highest increases in antioxidant capacity and phenolic content (up to 30% higher in FRAP), while Celluclast^®^ 1.5 L generated the highest rise in soluble dietary fiber (up to 2.75 g/100 g) and the best sensory acceptance. Moreover, Celluclast^®^ 1.5 L significantly modified starch digestibility, reducing rapidly digestible starch by 14% and increasing slowly digestible starch by over 150%, suggesting a lower predicted glycemic response. Conclusions: Incorporating the enzyme-treated artichoke and broccoli by-products into GF breads effectively enhances soluble fiber, antioxidant potential, and sensory quality. Among treatments, Celluclast^®^ 1.5 L applied to artichoke proved most effective overall, providing a balanced improvement in nutritional and functional attributes. These findings revealed the potential of Celluclast^®^ 1.5 L-treated artichoke by-products as a source of natural bioactive compounds for developing clean-label, nutritionally enhanced GF breads.

## 1. Introduction

Celiac disease (CD) is a chronic autoimmune disorder triggered by the consumption of gluten, a protein found in cereals such as wheat, barley, and rye, in individuals who are genetically predisposed. This condition leads to inflammation and damage to the villi of the small intestine, resulting in malabsorption of nutrients and subsequent nutritional deficiencies [1]. Currently, a gluten-free (GF) diet is the only treatment that has been shown to produce positive results in these patients. In recent decades, the incidence of celiac disease has increased, and together with the strict dietary requirement, this has driven a growing demand for GF products on the market [2,3]. Despite the therapeutic efficacy of a GF diet, commercial GF products often contain low levels of dietary fiber, minerals such as zinc, magnesium, and iron, and vitamins (particularly B vitamins), while being high in calories, fats, and sugars [4]. These nutritional imbalances in commercial GF products can increase the risk of nutritional deficiencies and metabolic disorders in individuals who rely on them [5].

Several innovative approaches have recently emerged to enhance the nutritional value of GF products. One of the most relevant developments is the use of pseudocereals such as quinoa, amaranth, and buckwheat, as well as legume flours, which are rich in high-quality protein, dietary fiber, minerals, and bioactive compounds [6,7]. Similarly, there has been growing interest in the inclusion of flax seeds due to their high omega-3 fatty acid, lignan, and soluble fiber content, which contribute to cardiovascular health and glycemic control [8,9]. Additionally, extracts from plants, such as grapes, bananas, apples, and carrots, which are rich in phenolic compounds and antioxidants, are being investigated as functional ingredients to enhance the nutritional value and confer additional bioactive properties to GF products [10,11,12].

Food loss and waste represent a global challenge, with nearly one-third of food produced for human consumption discarded annually, resulting in inefficient resource use and considerable environmental impacts [13]. Within the framework of a circular economy, the valorization of vegetable by-products emerges as a sustainable strategy to mitigate these impacts while enabling the development of innovative products with enhanced nutritional value [14].

In this context, broccoli (*Brassica oleracea* var. *italica*) and artichoke (*Cynara scolymus* L.), edible inflorescence vegetables, are of great interest due to their high global production and numerous nutritional benefits. Broccoli production, whose combined output with cauliflower exceeded 26 million tons in 2023 [15], generates a considerable quantity of by-products, since the edible fraction accounts for only about 15% of the total weight, while the remaining parts—mainly stems and leaves—are commonly discarded post-harvest [16]. However, these non-edible fractions represent a valuable source of bioactive compounds, including glucosinolates and isothiocyanates, phenolic compounds, carotenoids, vitamin C, dietary fiber, and minerals [17]. These components have been widely associated with a range of health-promoting biological activities, such as anticarcinogenic, anti-inflammatory, antimicrobial, antioxidant, and antihypertensive effects [18]. Similarly, artichoke production reaches approximately 1.6 million tons annually [15], of which only the immature inflorescences are consumed, while the remaining 60–85%, primarily composed of outer bracts, leaves, and stems, is discarded as by-products [19]. In general, these by-product fractions are rich in nutrients and bioactive compounds of considerable interest, including phenolic acids derived from caffeoylquinic acid, flavonoids such as luteolin and apigenin, minerals, vitamins, and especially a high content of dietary fiber, particularly inulin and pectin [20,21]. Such compounds have been linked to antioxidant, anti-inflammatory, antimicrobial, anticancer, and prebiotic activities [22].

Although vegetables are rich in fiber, the insoluble fraction predominates, which is less fermentable and generates fewer health-promoting metabolites than the soluble fraction (SDF) [23]. Different techniques and methods, such as mechanical degradation, chemical treatments, thermal processes, and enzymatic methods, have been investigated to enhance the soluble fibers in foods [24]. Among these, enzymatic methods using cellulases, pectinases, and xylanases are particularly effective, operating under mild and environmentally friendly conditions [25,26].

Considering the limited literature on exploring the use of enzyme-treated artichoke and broccoli by-products as natural functional ingredients in gluten-free breads, this study investigated their effect on the physico-chemical, nutritional, and sensory properties of gluten-free formulations. The enriched breads were compared with a control formulation and with commercial gluten-free bread to assess improvements in fiber content, antioxidant potential, and overall quality.

In this study, GF bread formulations were developed with the inclusion of broccoli and artichoke by-products after enzymatic treatment, which were compared with the Control and commercial GF bread. The effects of these vegetable by-products and enzymatic treatment on the nutritional quality and sensory attributes of the breads obtained were evaluated. This approach aims not only to enhance the functional properties of GF products for celiacs but also to contribute to the sustainable management of agro-industrial waste.

## 2. Results and Discussion

### 2.1. Chemical Composition and Physicochemical Characteristics

The chemical composition, antioxidant capacity, and total phenolic compounds (TPC) of the different GF bread samples, including formulated breads and the commercial bread, are detailed in Table 1.

Comparative analyses were performed using the Control as a reference to determine the effects of incorporating by-products into the formulation. On the other hand, the experimental GF breads were compared with a commercial product to establish nutritional differences relative to a market-available option. In terms of macronutrients, the broccoli samples (BB and BBE) showed no significant differences compared to the Control, maintaining similar profiles in energy, fat, protein, moisture, ash, and carbohydrates. The only exception was a slight increase in protein content in BBE, although it was not statistically significant. In contrast, samples with artichoke by-products showed a significant decrease in fat levels (about 8 g/100 g) compared to the Control (12.33 g/100 g). The observed decrease in fat content can be attributed to the fat-binding capacity of dietary fiber, which has been demonstrated to retain or immobilize lipids within the food matrix, thereby limiting their extractability during analysis [27,28]. In addition, both BA and BAE showed a significant increase in the ash fraction, 5.46 and 4.82 g/100 g, respectively, compared to 2.38 g/100 g for the Control. The increase in ash content observed after adding artichoke extract can be attributed to the naturally high mineral content of this vegetable [29]. Similar findings were reported by Dadali, who observed higher ash levels in cakes enriched with artichoke by-product powder [30].

With respect to dietary fiber (DF) content, the untreated breads (BB and BA) showed differences compared to the Control. BB exhibited similar insoluble (IDF, 10.68 g/100 g) and total fiber (TDF, 11.22 g/100 g) levels as the Control, whereas BA showed markedly higher IDF and TDF (17.05 and 18.58 g/100 g, respectively). Breads subjected to enzymatic treatment (BBE and BAE) displayed a significant increase in the soluble fiber fraction (SDF), with BBE reaching 2.16 g/100 g and BAE reaching the highest value of 2.75 g/100 g. However, IDF and TDF in the treated breads were slightly lower than in their respective untreated counterparts due to the conversion of insoluble fiber into soluble fiber, while remaining elevated compared to the Control. Similar results were observed in a previous study, where artichoke extracts generally had higher levels of dietary fiber than broccoli extracts, and when different enzymatic treatments were applied, their SDF levels increased significantly while reducing IDF levels [31]. In the case of artichoke, the commercial enzyme Celluclast^®^ 1.5 L was used, which catalyzes the hydrolysis of β-1,4-glycosidic bonds in insoluble polysaccharides, resulting in the release of water-soluble polysaccharides and oligosaccharides [32]. On the other hand, the findings in the study of the enzymatic treatment of broccoli by-products are attributed to the use of Viscozyme^®^ (an enzyme complex composed of arabinase, cellulase, β-glucanase, hemicellulase, and xylanase). This treatment is justified by the predominant presence of cellulose, xylans, and pectins in the wall of broccoli stems, which, when fragmented, release soluble oligosaccharides and, consequently, increase the SDF [33].

Additionally, the analyses revealed significant variations in antioxidant capacity (FRAP, ABTS, and DPPH) and total phenolic content (TPC) among the evaluated samples. All bread supplemented with vegetal extracts exhibited improvements compared to the Control. Treatment with both commercial enzymes caused significant changes (*p* < 0.05). In the GF bread with broccoli, enzymatic treatment resulted in increases of 30.9%, 37.3%, 29.7%, and 28.9% for FRAP, ABTS, DPPH, and TPC, respectively. In contrast, enzymatic treatment in artichoke bread produced more moderate changes, with increases of 9.3%, 27.6%, and 17.3% in ABTS, DPPH, and TPC, respectively. The differences observed between breads enriched with broccoli and artichoke extracts and the Control can be attributed to the higher content of polyphenols and other bioactive compounds in these plant matrices [34,35,36,37,38,39]. However, despite their abundance, the thermal processing involved in bread-making can partially degrade these compounds. As reported by Franke et al., the retention of bioactive compounds is highly dependent on processing conditions, which may explain why the enriched breads did not show markedly higher antioxidant activity or phenolic content compared to Control [40]. However, thermal processing during baking may reduce some heat-sensitive compounds, attenuating the beneficial effect of these extracts. On the other hand, the increases observed in antioxidant capacity and TPC in BBE and BAE can be attributed to the enzymatic action on the plant matrix. This action degrades cell wall components, releasing bound polyphenols, which are then transformed into soluble and bioavailable forms. Additionally, enzymatic hydrolysis generates oligosaccharides with inherent antioxidant activity, further contributing to the overall effect [31,41,42].

BC, predominantly composed of refined flours, starches, and processed vegetable fats, contains lower amounts of total dietary fiber (4.07 g/100 g), protein (2.90 g/100 g), and antioxidant capacity, together with a higher carbohydrate content (43.48 g/100 g) compared to the control formulation. In contrast, the Control bread, made with buckwheat flour, vegetable proteins, and seeds, incorporates highly valuable ingredients that provide it with a clearly superior nutritional and functional profile [43,44,45].

Recent advances in enzyme-assisted technologies have significantly improved the value of agro-industrial by-products for functional food development. Innovative approaches such as ultrasound-assisted enzymatic hydrolysis and high-pressure–enzyme combinations have been shown to enhance enzyme accessibility to plant cell wall polysaccharides, increasing the release of soluble dietary fiber and bound phenolics [46,47,48]. In parallel, enzyme engineering has yielded cellulases and multi-enzyme complexes with superior thermal stability and catalytic efficiency, broadening their industrial applicability [49]. Despite these advances, their implementation can be limited by cost and complexity. Our findings demonstrate that commercially available enzyme preparations applied under mild conditions effectively enhance soluble fiber content and antioxidant capacity in gluten-free breads enriched with artichoke and broccoli by-products.

### 2.2. Starch and Sugar Content

Table 2 shows the results for the different fractions of digestible starch: slow (SDS), rapid (RDS), and total (TDS); and resistant starch (RS). The profile of the samples in terms of monosaccharides and disaccharides is also detailed.

The different bread formulations showed significant variations in starch digestibility and simple sugar profile (*p* < 0.05). BC had a significantly higher RDS content than Control (51.96 and 44.20 g/100 g, respectively) and a lower SDS concentration (4.83 and 7.17 g/100 g). Additionally, it presented the lowest RS content among the samples (1.04 g/100 g). This profile corresponds to that described by Giuberti and Gallo, who reported that commercially available GF products tend to contain highly available starches and a low resistant fraction due to refined flours and aerated structure, resulting in a higher glycemic index [50]. In contrast, Control showed a more favorable profile, consistent with Di Cairano et al., who reported that less refined or underutilized flours, such as buckwheat flour used in this study, reduce rapid starch digestion and glycemic response [51].

Conversely, formulations containing broccoli and artichoke extracts, particularly those treated enzymatically (BBE and BAE), showed a significant reduction in RDS (41.11 and 38.00 g/100 g, respectively), and a marked increase in SDS (21.72 and 18.23 g/100 g). The RS content remained similar among the different formulations, ranging from 1.16 to 1.58 g/100 g, with BBE and BAE once again showing the highest values. These findings suggest that the incorporation of plant extracts, and especially their enzymatic treatment, significantly modifies the digestibility of starch in GF breads. A redistribution of the starch fractions towards a slower profile was observed, which could imply a lower glycemic index of the final product [50]. The lower proportion of RDS and the increase in SDS in breads enriched with plant extracts are consistent with the findings of Santamaría et al. and Melini et al., who reported similar effects when incorporating different flours or plant extracts into GF breads [52,53]. The observed effect was attributed to the presence of phenolic compounds and plant fibers, which interfere with starch and modify its gelatinization. Consequently, the broccoli and artichoke extracts used in this study, rich in indigestible polysaccharides and antioxidants, would have contributed to a bread matrix less accessible to digestive enzymes. Moreover, the more pronounced effects in the enzyme-treated samples can be explained by mechanisms similar to those reported by Kasprzak et al., where enzyme treatment of different starch sources modified their molecular structure, increasing the proportion of RS and reducing rapid digestibility [54]. All this suggests that the combination of plant extracts and enzymatic treatment may be an effective strategy for improving the digestion profile of starch in GF breads.

The sugar profile showed variations consistent with starch behavior. BC presented the highest glucose and maltose content (0.80 and 1.31 g/100 g, respectively), significantly higher than those of Control. This reflects faster hydrolysis and greater availability of simple sugars due to its composition of refined flours, added sugar, and glucose syrup. On the other hand, the incorporation of artichoke and broccoli extracts significantly modified the sugar profile compared to the Control. Bread containing broccoli exhibited a moderate increase in fructose and glucose levels, while those containing artichoke demonstrated higher concentrations, particularly in BAE, which exhibited a greater release of fructose and glucose. These effects can be explained by the release of intrinsic sugars from the plant matrices and by the partial hydrolysis of polysaccharides during enzymatic treatment [53,55]. Therefore, the observed increase in soluble sugars in these samples does not necessarily indicate greater starch digestibility, but rather the incorporation of simple carbohydrates from the functional ingredients.

### 2.3. Color and Acidity Profile

Table 3 presents the color and acidity parameters measured for the different GF breads analyzed in this study.

Regarding color measurements (CIEL*a*b*), the addition of broccoli and artichoke extracts, as well as their enzymatically treated extracts, produced significant changes in the color of bread compared to the Control. The incorporation of these extracts led to a decrease in L* and an increase in *C* and b*, resulting in darker breads with greater color intensity and yellowish tones. Enzymatic treatment further enhanced these effects, significantly decreasing L* and increasing *C*, a*, and b*, yielding breads that were darker, with more intense colors and pronounced reddish and yellowish tones. These changes can be attributed to the pigments present in the plant matrices, such as chlorophylls and carotenoids. Previous studies have shown that the use of pigmented plant by-products, such as broccoli or artichoke, in bakery formulations affects color parameters, often resulting in strong green or yellowish hues in the final product [56,57,58,59]. An important factor to consider in the development of new products is the total color difference (∆E*) relative to the Control, with values above 3 being perceptible to the human eye. In this study, all reformulated breads showed ∆E* values ranging from 5.32 to 15.18, indicating noticeable color changes. Similar observations have been reported in studies using pigmented plant materials, such as green algae (*Chlorella vulgaris*), which also induced significant color modifications in breads [60]. BC was significantly lighter and more yellow than the Control, with higher saturation and hue, resulting in a noticeably brighter appearance. These differences can be attributed to its composition of refined, yellowish flours, whereas the Control, made with buckwheat flour and flax seeds, exhibits a darker, less saturated color.

In terms of acidity, BC exhibited a lower pH than Control, but its total titratable acidity (TTA) and levels of organic acids (L-lactic and acetic) were lower. This finding indicates that the acidity present in commercially available bread is not a consequence of yeast fermentation; rather, it is derived from other technological adjustments incorporated during its industrial formulation. Conversely, the incorporation of broccoli and artichoke extracts resulted in significant increases in TTA, which were more pronounced in enzymatically treated samples (6.41 and 6.82 mL NaOH/10 g, in BBE and BAE, respectively). This reflects a more intense fermentation process, increasing organic acids, particularly acetic acid. Consistent with the TTA results, the samples that exhibited higher concentrations were BBE and BAE, which presented 2.04 and 2.47 g acetic acid/kg of sample. These results can be attributed to enzymatic hydrolysis, which releases sugars and phenolic compounds from plant cell walls that serve as substrates for microbial fermentation during bread making. Recent studies have also shown that the fermentation of fiber- and protein-rich plant by-products with lactic acid bacteria significantly increases TTA and the concentration of organic acids [61]. These findings demonstrate that the synergistic action of plant extracts and enzymatic treatments effectively stimulates microbial activity, enhancing acid production in bread [62].

### 2.4. Sensory Analysis of Gluten-Free Breads

**Figure 1:**
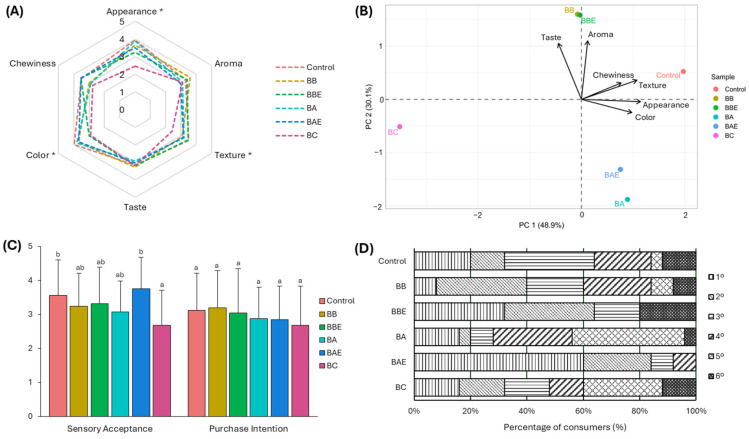
Sensory analysis of gluten-free breads formulations. Radar plots showing the sensory attributes of the different formulations (**A**). Principal component analysis (PCA) (**B**) biplot illustrating the distribution of gluten-free samples according to their sensory profiles and the contribution of each attribute to sample differentiation. Different letters (a, b) indicate statistically significant differences between samples (* *p* < 0.05). Sensory acceptance and purchase intention (**C**), and preference ordination (**D**) of gluten-free bread samples. Control: Bread without extract; BB: Bread with broccoli; BBE: Bread with enzymatically treated broccoli; BA: Bread with artichoke; BAE: Bread with enzymatically treated artichoke; BC: Commercial bread.

Figure 1 presents the results of the sensory evaluation and consumer acceptance of the GF bread formulations, assessed by a trained panel. In the sensory attribute analysis (Figure 1A), significant differences were observed in appearance, color, and texture (*p* < 0.05). The BC formulation obtained lower scores for appearance and color, whereas the other formulations enriched with broccoli and artichoke extracts exhibited sensory profiles comparable to the Control. To further explore the relationships among samples, a principal component analysis was conducted (Figure 1B). The biplot indicated that the Control bread was primarily associated with texture and appearance, while the BB and BBE formulations were positively associated with aroma and taste. In contrast, the BC formulation was clearly differentiated from the other treatments, confirming its less favorable sensory profile.

Regarding overall acceptance and purchase intention (Figure 1C), no significant differences were observed in the latter variable. However, significant differences in overall acceptance were detected (*p* < 0.05), with the Control and BAE formulations achieving the highest scores, while BC recorded the lowest values. Similar improvements in sensory acceptability have been reported for GF bakery products enriched with plant extracts, including *Moringa Oleifera*, *Linum usitatissimum*, *Citrus × sinensis*, and *Daucus carota* [63,64,65,66].

Finally, the preference ranking analysis (Figure 1D) revealed that the BBE and BAE formulations were the most highly valued by consumers, as they concentrated the highest percentages in the top positions. Conversely, the BC and BA formulations were predominantly ranked in the lowest positions, consistent with their lower acceptance levels.

### 2.5. Pearson Correlations

**Figure 2:**
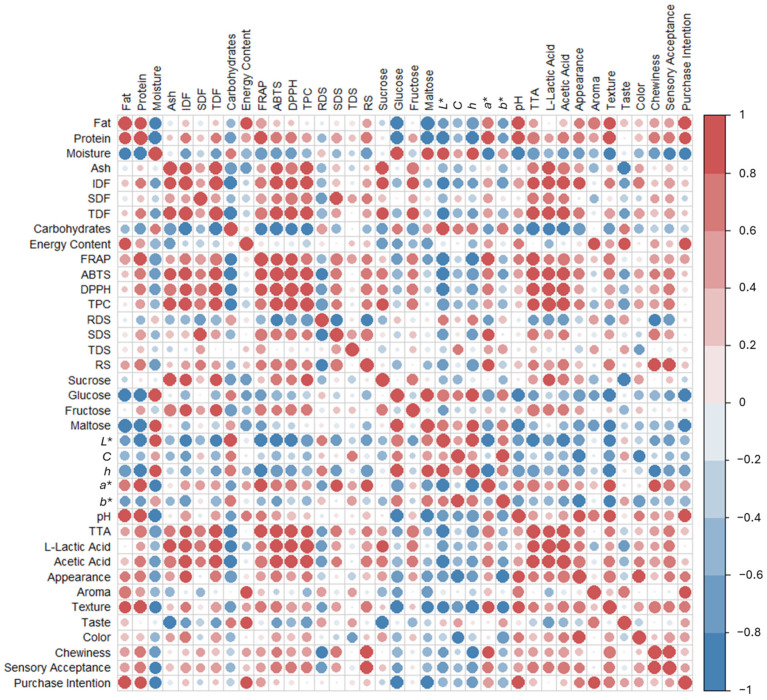
Pearson correlation heatmap between different measured parameters. Circle size represents the magnitude of the correlation coefficient, with larger circles indicating stronger correlations. IDF: Insoluble dietary fiber; SDF: Soluble dietary fiber; TDF: Total dietary fiber; TPC: Total phenolic content; RDS: Rapidly digestible starch; SDS: Slowly digestible starch; TDS: Total digestible starch; RS: Resistant starch; TTA: total titratable acidity.

The results obtained for proximal composition, antioxidant activity, physicochemical properties, and sensory characteristics were subjected to a Pearson correlation analysis (Figure 2) to verify similarities and relationships between the attributes. The Pearson correlations of the different breads showed a positive correlation between fat and protein with purchase intention (r = 0.97 and r = 0.80, respectively). This association is to be expected, as fat contributes softness, juiciness, and better texture to the crumb, in addition to intensifying the flavor. On the other hand, protein contributes to better structure, volume, and elasticity [67]. This may explain the low results of BC compared to handmade breads, which had better nutritional values and better acceptance. These results are corroborated by the correlation between texture (r = 0.74) and chewiness (r = 0.88) and the sensory acceptance of sourdough breads, attributes in which BC obtained the lowest results compared to the other breads analyzed.

On the other hand, strong positive correlations were found between IDF, TDF, and SDF and the results obtained in antioxidant activity (ABTS, FRAP, and DDPH) and TPC. These associations indicate that breads with higher fiber fractions also had higher levels of bioactive compounds, confirming that broccoli and artichoke extracts had an impact on both the fiber fortification of GF breads and a higher content of bioactive compounds. Moreover, this positive relationship can be attributed to the ability of dietary fiber, particularly the insoluble fraction, to interact with phenolic compounds through both covalent and non-covalent bonds, thereby acting as a binding matrix for these bioactive molecules [68]. Furthermore, breads with enzymatically treated extracts (BAE and BBE) showed higher antioxidant values than their corresponding analogs (BA and BB), suggesting that enzymatically treated fiber may facilitate the release of bound phenols during extraction and analysis.

## 3. Materials and Methods

### 3.1. Broccoli and Artichoke Extracts Preparation

Agro-industrial by-products from broccoli (stems and leaves) and artichoke (stems, leaves, and external bracts) were supplied by Cricket (Lorca, Spain). All by-product powders were obtained following the method described by Ayuso et al. [31] The different broccoli and artichoke bio-residues were dried in a forced-air oven at 50 °C for 24 h. Once completely dried, the by-products were ground and sieved to produce fine powders with a particle size of 500 µm.

Viscozyme^®^ L, a multi-enzyme complex (cellulase, arabanase, β-glucanase, and xylanase) with strong pectolytic activity from *Aspergillus aculeatus*, and Celluclast^®^ 1.5 L, a cellulase obtained from *Trichoderma reesei*, were utilized for the enzymatic treatment of broccoli and artichoke by-products, respectively. Both enzymes were purchased from Novozymes (Bagsværd, Denmark).

For the enzymatic treatment, diluted broccoli and artichoke powders (1:5, *w*/*v*) were incubated with Viscozyme^®^ L (pH 4.5, 45 °C) and Celluclast^®^ 1.5 L (pH 6, 50 °C), respectively, at 0.45% (*v*/*w*) for 24 h under continuous magnetic stirring. The hydrolyzed mixtures were dried at 60 °C in a forced-air oven and subsequently milled again to achieve the same particle size as before enzymatic treatment.

### 3.2. Gluten-Free Breads Elaboration

The ingredients used in the GF bread formulation, as detailed in Table 4, were obtained from a local supermarket in Murcia, Spain. Ground flax seeds, sugar, and dry yeast were mixed with water and allowed to stand for 1 h at 25 °C to activate the yeast and form the flaxseed gel, thereby supporting proper fermentation and improving dough cohesion. The remaining bread ingredients were then added and mixed to obtain a fluid and homogeneous dough using an automatic bread machine (Silvercrest^®^, IAN 285058, Lidl Stiftung & Co. KG, Neckarsulm, Germany) set to the “gluten-free” program. This program comprises multiple stages of kneading, resting, and baking, with a total duration of 2 h.

Five different GF formulations were developed: a control bread without any vegetable extract (Control); breads enriched with 12.5 g of dried broccoli extract, either untreated (BB) or enzymatically treated (BBE); and breads containing 8.8 g of dried artichoke extract, either untreated (BA) or enzymatically treated (BAE). After baking, the loaves were allowed to cool for 1 h, then sliced and vacuum-packed in polyethylene bags and stored at −20 °C until further analysis. The amounts of extract and broccoli were estimated based on the fiber content of all ingredients to ensure that the breads could be declared as “high fiber” products in accordance with Regulation (EC) No 1924/2006 [69].

**Figure 3:**
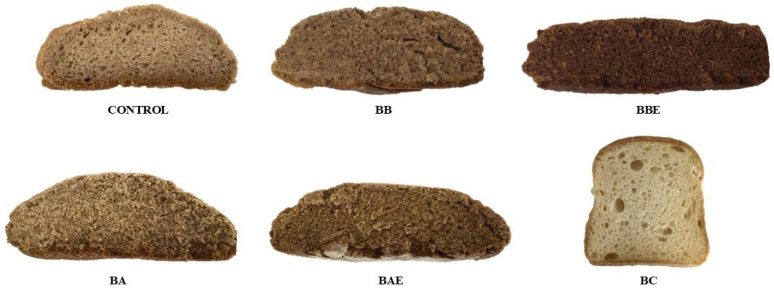
Visual appearance of the different gluten-free bread formulations. Control: Bread without extract; BB: Bread with broccoli; BBE: Bread with enzymatically treated broccoli; BA: Bread with artichoke; BAE: Bread with enzymatically treated artichoke; BC: Commercial bread.

For comparison, the five GF formulations were evaluated alongside a commercial GF bread (BC) purchased from a local supermarket. According to the product label, its composition includes: water, maize starch, rice flour, sugar, eggs (11%), vegetable margarine (palm fat, coconut fat, water, rapeseed oil, salt; emulsifier: mono- and diglycerides of fatty acids; natural flavor), glucose syrup, milk powder, vegetable fiber (psyllium), thickeners (hydroxypropyl methylcellulose, guar gum), yeast, emulsifier (mono- and diglycerides of fatty acids), salt, acidifying agent (tartaric acid), and natural vanilla flavor. The visual appearance of the GF breads is presented in Figure 3.

### 3.3. Proximal Composition

The proximal composition of formulated and commercial GF breads was analyzed according to the official protocols set forth by the Association of Official Analytical Chemists (AOAC) employing the following methods: moisture (964.22), ash (923.03), protein (955.04), total fat (920.39), and dietary fiber fractions (insoluble, soluble, and total) (985.29) [70]. The carbohydrate content was calculated by difference, based on the values obtained for moisture, protein, fat, and ash [71]. The energy content was estimated using standard conversion factors, in accordance with the Food and Agriculture Organization of the United Nations (FAO) recommendations [72].

### 3.4. Starch Determination

Rapidly digestible starch (RDS), slowly digestible starch (SDS), total digestible starch (TDS), and resistant starch (RS) were determined using the enzymatic Digestible and Resistant Starch Assay Kit (Megazyme International, Bray, Ireland), following the procedure recommended by the manufacturer. This standardized and widely used method is based on the Englyst et al. approach and involves a kinetic enzymatic digestion coupled with colorimetric quantification of glucose released at 510 nm wavelength [73]. Enzymatic digestion was conducted at 37 °C for 240 min using pancreatic alpha-amylase and amyloglucosidase (PAA/AMG). Aliquots were collected at 20 min to quantify RDS, at 120 min for SDS, and at 240 min to determine TDS and RS. Results were expressed as g of starch per 100 g of bread.

### 3.5. Physicochemical Parameters

Color was assessed using a CR-400 portable colorimeter (Konica Minolta, Tokyo, Japan), calibrated with a standard white reference plate. The measured parameters included lightness (L*), red-green component (a*), yellow-blue component (b*), chroma (*C*), and hue (h) following the CIEL*a*b* color space system. Measurements were conducted in triplicate on 16 mm-thick slices from each bread sample. The total color difference (∆E*) of each formulation compared to the Control bread was calculated with the following formula:


$${∆E}^{*}=\sqrt{{\left(L_{s}^{*}-L_{c}^{*}\right)}^{2}+{\left(a_{s}^{*}-a_{c}^{*}\right)}^{2}+{\left(b_{s}^{*}-b_{c}^{*}\right)}^{2}}$$

The pH was determined potentiometrically using a sensION+ PH31 pH meter (Hach-Lange, Barcelona, Spain) in a 1:10 (*w*/*v*) homogenized suspension of bread in distilled water. Total titratable acidity (TTA) was then assessed in the same suspension by titration with 0.1 N sodium hydroxide (NaOH) until the pH reached 8.5. Results were expressed as mL NaOH/10 g.

L-lactic acid and acetic acid concentrations were quantified using specific commercial enzymatic assay kits (BioSystems S.A., Barcelona, Spain), according to the manufacturer’s instructions. Assays were performed on the BioSystems Y15 automatic analyzer (BioSystems S.A., Barcelona, Spain), measuring absorbance at 340 nm. Results were expressed as grams of L-lactic or acetic acid per kilogram of bread.

### 3.6. Determination of Disaccharides and Monosaccharides

Monosaccharides (glucose and fructose) and disaccharides (sucrose and maltose) concentrations were analyzed using enzymatic methods with three commercial kits (BioSystems S.A., Barcelona, Spain). Measurements were carried out spectrophotometrically at 340 nm using a BioSystems Y15 automatic analyzer (BioSystems S.A., Barcelona, Spain). Results were expressed as g of glucose, fructose, sucrose, or maltose per 100 g of bread.

### 3.7. Antioxidant Capacity and Total Phenolic Content (TPC) Analysis

Before analysis, bioactive compounds were extracted from the breads by homogenizing 2 g of sample with a 1:5 methanol/water (80:20, *v*/*v*) solution. The mixtures were incubated for 24 h at 4 °C in the dark, then centrifuged at 4500 rpm for 25 min at 4 °C. The supernatants were filtered through 0.45 µm nylon filters and stored at −20 °C until analysis.

The antioxidant capacity of the samples was evaluated using three complementary spectrophotometric assays. The ferric reducing antioxidant power (FRAP) assay was carried out by combining 100 µL of the extracted sample with 1 mL of FRAP reagent, which contained acetate buffer, FeCl_3_·6H_2_O, and 2,4,6-tripyridyl-s-triazine (TPTZ) [74]. The absorbance was measured at 593 nm after 4 min in the dark. For the DPPH free radical scavenging 100 µL of the sample was mixed with 3.9 mL of a methanolic 2,2-diphenyl-1-picrylhydrazyl solution and incubated in dark conditions for 30 min [75]. The decrease in absorbance at 515 nm was recorded. The ABTS radical cation decolorization assay was conducted by incubating 100 µL of the sample with 2,2’-azino-bis (3-ethylbenzothiazoline-6-sulfonic acid) for 4 min, followed by measuring absorbance at 734 nm [76]. All antioxidant activity assays were performed in triplicate, and results expressed as µmol trolox equivalents (TE) per g of sample. In addition, the TPC of the bread samples was determined following the method described by Singleton et al., by measuring the absorbance at 750 nm after a 1-h incubation of the extracted sample mixed with the Folin–Ciocalteu reagent [77]. The analysis was carried out in triplicate, and results were expressed as mg gallic acid equivalents (GAE) per g of sample. All spectrophotometric measurements were performed on an Evolution 300 spectrophotometer (Thermo Scientific, Waltham, MA, USA).

### 3.8. Sensory Analysis

A hedonic sensory evaluation was conducted on the six GF bread samples using a structured 5-point scale to assess sensory attributes, including appearance, aroma, texture, taste, color, chewiness, overall acceptability, and purchase intention. The bread samples were cut into 3 cm^3^ portions and coded with randomly selected three-digit numbers. The sensory panel consisted of 25 trained assessors (14 female, 11 male) aged between 18 and 60 years. Participants were required to be healthy adults without sensory impairments or food allergies related to the tested products. Exclusion criteria included smoking and medical conditions that could affect sensory perception. The evaluation was conducted in individual sensory booths according to ISO guidelines [78], with mineral water provided for rinsing their mouths between samples.

### 3.9. Statistical Analysis

All determinations were conducted in three independent experimental replicates (*n* = 3), and results were expressed as mean ± standard deviation (SD). Statistical analysis was performed using the SPSS software, version 28.0 (IBM Corporation, Armonk, NY, USA). Differences among GF breads were determined by one-way analysis of variance (ANOVA), setting a significance threshold of *p* < 0.05, followed by Tukey’s post hoc test. The datasets from all experimental analyses were integrated and visualized through a heatmap of Pearson correlation coefficients, constructed using the ‘pheatmap’ package (version 2.5.0) in R software (version 4.5.0) (R Foundation for Statistical Computing, Vienna, Austria).

## 4. Conclusions

The present study expands current knowledge on the use of enzyme-treated vegetable by-products as natural functional ingredients in gluten-free bakery formulations. Specifically, the effects of enzymatically treated artichoke and broccoli extracts were assessed as sustainable alternatives to synthetic additives in gluten-free breads. The incorporation of the by-products represents a sustainable and effective strategy for developing gluten-free (GF) breads with enhanced nutritional, functional, and sensory properties compared with a commercial reference. Among the enzymatic treatments, Celluclast^®^ 1.5 L applied to artichoke by-products (BAE) exhibited the most significant overall improvements. This enzyme increased soluble dietary fiber up to 2.75 g/100 g, while markedly modifying starch digestibility by reducing the rapidly digestible fraction by 14% and increasing the slowly digestible fraction by more than 150%, suggesting a lower predicted glycaemic response. Although Viscozyme^®^ L mainly enhanced antioxidant capacity (30% in FRAP and 37% in ABTS), Celluclast^®^ 1.5 L achieved a more balanced enhancement across nutritional, functional, and sensory attributes, with BAE showing the highest consumer acceptance.

In contrast, non-treated extracts exerted limited influence on antioxidant activity and product quality. The enzymatic treatment effectively improved the release of bioactive compounds and mitigated undesirable effects on crumb color and structure, demonstrating greater functional performance than the control formulation and the commercial reference bread.

Correlations between compositional and sensory parameters indicated that increased soluble fiber and phenolic content were associated with improved sensory perception and antioxidant performance. Overall, the findings demonstrate the potential of enzyme-treated artichoke and broccoli by-products as sustainable, clean-label ingredients for the formulation of nutritionally improved GF breads, contributing to the valorization of agro-industrial residues and the advancement of circular food production systems. Further research is required to evaluate their impact on oxidative stability and storage performance under industrial conditions.

## Figures and Tables

**Table 1:** Proximal composition, antioxidant activity, and total phenolic content (TPC) of gluten-free breads.

	Control	BB	BBE	BA	BAE	BC
Proximal composition						
Energy content (kcal/100 g)	287.70 ± 5.66 a	287.56 ± 3.05 a	280.26 ± 4.90 a	209.22 ± 3.51 c	232.40 ± 0.66 b	235.70 ± 4.51 b
Fat (g/100 g)	12.33 ± 0.99 a	12.10 ± 0.44 a	11.90 ± 0.66 a	8.45 ± 1.34 b	8.08 ± 0.18 b	5.58 ± 0.15 b
Protein (g/100 g)	12.14 ± 0.58 a	12.88 ± 3.28 a	15.03 ± 0.71 a	10.61 ± 0.30 a	11.13 ± 0.47 a	2.90 ± 0.06 b
Moisture (g/100 g)	29.89 ± 1.44 c	27.64 ± 0.16 c	29.30 ± 0.31 c	34.22 ± 0.27 b	29.79 ± 0.10 c	42.37 ± 1.03 a
Ash (g/100 g)	2.38 ± 0.01 b	2.42 ± 0.00 b	2.58 ± 0.03 b	5.46 ± 0.68 a	4.82 ± 0.18 a	1.61 ± 0.10 b
IDF (g/100 g)	10.68 ± 1.29 b	12.07 ± 0.24 b	10.77 ± 0.56 b	17.05 ± 0.42 a	14.65 ± 0.05 a	3.07 ± 0.01 c
SDF (g/100 g)	0.54 ± 0.01 c	1.11 ± 0.30 bc	2.16 ± 0.69 ab	1.52 ± 0.57 abc	2.75 ± 0.27 a	1.00 ± 0.02 bc
TDF (g/100 g)	11.22 ± 1.28 b	13.18 ± 0.05 b	12.92 ± 0.13 b	18.58 ± 0.15 a	17.40 ± 0.22 a	4.07 ± 0.01 c
Carbohydrates (g/100 g)	32.05 ± 0.23 b	31.79 ± 3.50 b	28.27 ± 0.47 bc	22.70 ± 2.43 c	28.79 ± 0.23 bc	43.48 ± 0.85 a
Antioxidant activity and TPC						
FRAP (µmol TE/g)	13.73 ± 0.72 b	18.43 ± 2.75 ab	24.12 ± 0.97 a	19.27 ± 3.30 ab	18.92 ± 0.67 ab	5.72 ± 0.70 c
ABTS (µmol TE/g)	27.65 ± 1.36 cd	23.95 ± 1.33 d	32.88 ± 3.03 bc	37.05 ± 1.11 ab	40.48 ± 2.18 a	7.67 ± 1.11 e
DPPH (µmol TE/g)	8.38 ± 0.76 bc	11.29 ± 2.69 ab	14.64 ± 1.44 ab	15.17 ± 2.30 ab	19.35 ± 3.53 a	1.88 ± 1.18 c
TPC(mg GAE/g)	7.72 ± 0.14 b	7.05 ± 1.12 b	9.09 ± 0.29 b	12.28 ± 0.01 a	14.40 ± 0.64 a	2.98 ± 0.02 c

a–e: Different letters within the same row indicate statistically significant differences between samples (*p* < 0.05). Control: Bread without extract; BB: Bread with broccoli; BBE: Bread with enzymatically treated broccoli; BA: Bread with artichoke; BAE: Bread with enzymatically treated artichoke; BC: Commercial bread; IDF: Insoluble dietary fiber; SDF: Soluble dietary fiber; TDF: Total dietary fiber; FRAP: Ferric reducing antioxidant power assay; ABTS: 2,2′-azino-bis(3-ethylbenzothiazoline-6-sulfonic acid) radical cation scavenging assay; DPPH: 2,2-diphenyl-1-picrylhydrazyl radical scavenging assay; TPC: Total phenolic content.

**Table 2:** Starch and sugar content of gluten-free breads (g/100 g).

	Control	BB	BBE	BA	BAE	BC
Digestible and resistant starch						
RDS	44.20 ± 0.78 b	53.25 ± 0.76 a	41.11 ± 0.08 c	44.97 ± 0.57 b	38.00 ± 0.01 d	51.96 ± 0.30 a
SDS	7.17 ± 0.46 d	7.15 ± 0.03 d	21.72 ± 0.23 a	10.23 ± 0.33 c	18.23 ± 0.33 b	4.83 ± 0.28 e
TDS	51.37 ± 1.24 c	60.41 ± 0.74 a	62.83 ± 0.30 a	55.19 ± 0.23 b	56.22 ± 0.35 b	56.79 ± 0.58 b
RS	1.42 ± 0.04 b	1.22 ± 0.05 c	1.48 ± 0.03 ab	1.16 ± 0.01 c	1.58 ± 0.01 a	1.04 ± 0.01 d
Disaccharides and monosaccharides						
Sucrose	0.15 ± 0.01 c	0.10 ± 0.01 cd	0.05 ± 0.01 de	0.58 ± 0.04 a	0.40 ± 0.03 b	0.02 ± 0.01 e
Glucose	0.11 ± 0.01 d	0.17 ± 0.01 d	0.14 ± 0.01 d	0.33 ± 0.02 c	0.41 ± 0.03 b	0.80 ± 0.03 a
Fructose	0.04 ± 0.01 d	0.20 ± 0.01 b	0.15 ± 0.01 c	0.28 ± 0.02 a	0.18 ± 0.02 bc	0.04 ± 0.01 d
Maltose	0.07 ± 0.02 d	0.09 ± 0.02 d	0.16 ± 0.03 d	0.61 ± 0.06 c	0.81 ± 0.02 b	1.31 ± 0.02 a

a–e: Different letters within the same row indicate statistically significant differences between samples (*p* < 0.05). Control: Bread without extract; BB: Bread with broccoli; BBE: Bread with enzymatically treated broccoli; BA: Bread with artichoke; BAE: Bread with enzymatically treated artichoke; BC: Commercial bread; RDS: Rapidly digestible starch; SDS: Slowly digestible starch; TDS: Total digestible starch; RS: Resistant starch.

**Table 3:** Color and acidity parameters of gluten-free breads.

	Control	BB	BBE	BA	BAE	BC
CIEL*a*b* parameters						
L*	55.15 ± 1.58 b	52.93 ± 0.82 b	41.02 ± 1.61 d	47.67 ± 2.39 c	47.19 ± 0.74 c	79.45 ± 0.51 a
*C*	12.54 ± 0.87 c	16.92 ± 0.71 b	18.01 ± 1.31 ab	13.52 ± 2.20 c	17.27 ± 0.43 ab	20.36 ± 0.55 a
*h*	66.94 ± 2.08 c	71.69 ± 0.14 b	61.36 ± 0.14 d	71.03 ± 1.27 b	69.49 ± 1.12 bc	86.27 ± 0.14 a
a*	5.32 ± 0.48 b	5.32 ± 0.19 b	8.63 ± 0.64 a	4.37 ± 0.53 c	6.05 ± 0.18 c	1.33 ± 0.05 a
b*	11.41 ± 0.76 d	16.06 ± 0.69 b	15.81 ± 1.14 bc	12.80 ± 2.17 cd	16.17 ± 0.52 b	20.31 ± 0.54 a
∆E*	-	5.32 ± 0.72 c	15.18 ± 2.98 b	8.25 ± 2.78 c	9.37 ± 0.92 c	26.20 ± 1.89 a
Acidity profile						
pH	5.92 ± 0.15 a	6.01 ± 0.21 a	5.76 ± 0.01 ab	5.66 ± 0.01 ab	5.48 ± 0.02 b	4.99 ± 0.06 c
TTA (ml NaOH/10 g)	4.74 ± 0.06 c	5.92 ± 0.16 b	6.41 ± 0.14 ab	6.22 ± 0.04 b	6.82 ± 0.05 a	3.08 ± 0.25 d
L-Lactic Acid (g/kg)	0.25 ± 0.01 b	0.24 ± 0.01 b	0.25 ± 0.01 b	0.34 ± 0.03 a	0.39 ± 0.01 a	0.15 ± 0.01 c
Acetic Acid (g/kg)	1.98 ± 0.03 c	1.94 ± 0.01 c	2.04 ± 0.01 c	2.26 ± 0.09 b	2.47 ± 0.01 a	1.20 ± 0.02 d

a–d: Different letters within the same row indicate statistically significant differences between samples (*p* < 0.05). CIEL*a*b*: International standard color space where L* represents lightness, a* the green–red axis, and b* the blue–yellow axis; Control: Bread without extract; BB: Bread with broccoli; BBE: Bread with enzymatically treated broccoli; BA: Bread with artichoke; BAE: Bread with enzymatically treated artichoke; BC: Commercial bread; TTA: total titratable acidity; ∆E*: Total color difference of each formulation compared to the control bread.

**Table 4:** Gluten-free breads formulation.

Ingredients	Control	BB	BBE	BA	BAE
Buckwheat flour (g)	125	125	125	125	125
Maize starch (g)	75	75	75	75	75
Lentil protein (g)	20	20	20	20	20
Ground flax seeds (g)	35.2	35.2	35.2	35.2	35.2
Dry yeast (g)	7	7	7	7	7
Water (g)	175	175	175	175	175
Salt (g)	4.2	4.2	4.2	4.2	4.2
Sugar (g)	4.2	4.2	4.2	4.2	4.2
Olive oil (mL)	25	25	25	25	25
Broccoli extract (g)	-	12.5	-	-	-
Enzyme-treated broccoli extract (g)	-	-	12.5	-	-
Artichoke extract (g)	-	-	-	8.8	-
Enzyme-treated artichoke extract (g)	-	-	-	-	8.8

Control: Bread without extract; BB: Bread with broccoli; BBE: Bread with enzymatically treated broccoli; BA: Bread with artichoke; BAE: Bread with enzymatically treated artichoke.

## Data Availability

The original contributions presented in this study are included in the article. Further inquiries can be directed to the corresponding author.
